# Fe‐CoP Electrocatalyst Derived from a Bimetallic Prussian Blue Analogue for Large‐Current‐Density Oxygen Evolution and Overall Water Splitting

**DOI:** 10.1002/advs.201800949

**Published:** 2018-08-14

**Authors:** Li‐Ming Cao, Yu‐Wen Hu, Shang‐Feng Tang, Andrey Iljin, Jia‐Wei Wang, Zhi‐Ming Zhang, Tong‐Bu Lu

**Affiliations:** ^1^ MOE Key Laboratory of Bioinorganic and Synthetic Chemistry School of Chemistry Sun Yat‐Sen University Guangzhou 510275 China; ^2^ Institute for New Energy Materials & Low Carbon Technologies School of Materials Science & Engineering Tianjin University of Technology Tianjin 300384 China; ^3^ Institute of Physics National Academy of Sciences of Ukraine Prospect Nauki 46 Kyiv 03028 Ukraine

**Keywords:** bifunctional electrocatalysts, large current density, oxygen evolution, Prussian blue analogues

## Abstract

Industrial application of overall water splitting requires developing readily available, highly efficient, and stable oxygen evolution electrocatalysts that can efficiently drive large current density. This study reports a facile and practical method to fabricate a non‐noble metal catalyst by directly growing a Co‐Fe Prussian blue analogue on a 3D porous conductive substrate, which is further phosphorized into a bifunctional Fe‐doped CoP (Fe‐CoP) electrocatalyst. The Fe‐CoP/NF (nickel foam) catalyst shows efficient electrocatalytic activity for oxygen evolution reaction, requiring low overpotentials of 190, 295, and 428 mV to achieve 10, 500, and 1000 mA cm^−2^ current densities in 1.0 m KOH solution. In addition, the Fe‐CoP/NF can also function as a highly active electrocatalyst for hydrogen evolution reaction with a low overpotential of 78 mV at 10 mA cm^−2^ current density in alkaline solution. Thus, the Fe‐CoP/NF electrode with meso/macropores can act as both an anode and a cathode to fabricate an electrolyzer for overall water splitting, only requiring a cell voltage of 1.49 V to afford a 10 mA cm^−2^ current density with remarkable stability. This performance appears to be among the best reported values and is much better than that of the IrO_2_‐Pt/C‐based electrolyzer.

## 1 Introduction

Developing renewable energy sources and reducing the environmental pollution are vital to the sustainable development of human beings.^1^ Water splitting has been considered as a promising technology to produce high‐purity hydrogen,^2^ a clean energy resource to meet the growing energy demand. However, the practical applications of electrocatalytic water splitting are very limited due to the high overpotentials of two half reactions, the hydrogen evolution reaction (HER) and the oxygen evolution reaction (OER), especially for large‐current‐density (>500 mA cm^−2^) OER.^3^ Hence, enormous research efforts have been devoted to explore water splitting electrocatalysts in order to reduce high overpotentials and prepare high‐efficiency electrocatalysts for HER and OER.^4^ At present, Pt‐based HER catalysts, and Ir‐based and Ru‐based OER catalysts, have been explored and shown impressive catalytic performance. Their high‐cost production and scarcity, however, are seriously limiting their large‐scale applications.^5^ Therefore, it is particularly desirable to design and develop non‐noble metal electrocatalysts for efficient water splitting.

Up to date, tremendous achievements have been acquired in the development of earth‐abundant element‐based electrocatalysts, including transition‐metal oxides/hydroxides,^6^ chalcogenides,^7^ phosphides,^8^ nitrides,^9^ and carbides.^10^ Among them, metal phosphides have been extensively investigated as high‐performance electrocatalysts for HER, showing low overpotential and small tafel slope in the acidic media.^11^ Recently, they were also reported as promising electrocatalysts for OER under the alkaline conditions.^12^ For example, NiCoP nanostructure and nanoporous Ni_2_P were both reported with outstanding catalytic performance for OER, and only required overpotentials of 280 and 200 mV to drive a 10 mA cm^−2^ current density in the alkaline conditions.^13^ Thus, the NiCoP can be used as bifunctional catalyst for overall water splitting, achieving a current density of 10 mA cm^−2^ at the cell voltage as low as 1.58 V. It is greatly appealing to efficiently drive overall water splitting by the same catalyst, as it can predigest the synthetic process and cut down the total costs.^14^ To date, it is still a challenging task, however a requisite work to explore low‐cost metal phosphides with low overpotentials for both HER and OER.

Large‐scale industrial application of electrocatalytic water splitting required the development of OER electrocatalysts that are low‐cost, robust, and can drive large current density (>500 mA cm^−2^) at low overpotentials (<300 mV). Up to now, there are few electrocatalysts that can meet the requirement, such as Ni‐Fe‐OH@Ni_3_S_2_/NF (nickel foam),[[qv: 15a]] NiFe/NF electrode,[[qv: 15b]] and Ni‐Fe‐S ultrathin nanosheets.[[qv: 15c]] To be used for industrial applications, the electrocatalytic performance should be further enhanced by design and preparation of electrocatalysts working well at large current densities to meet the strict criteria of industrial applications.^15^ Specifically, an ideal catalyst should behave the following characteristics: 1) abundant exposed active sites; 2) enhanced surface permeability; 3) superb electron conductivity; 4) high stability under strong oxidizing condition. Presently, most of the transition‐metal oxides and phosphides reported as the active catalysts are pulverous materials, which require costly conductive polymeric binders to prepare the electrodes. This obviously decreases the catalytic active sites and seriously affects the stability of the glued catalysts under high current densities as well as vigorous gas evolution conditions. In the large‐current‐density water splitting process, the effects of generated gas bubbles and the gas behavior are not negligible for releasing H_2_ and O_2_. One effective strategy for alleviating the above issues is direct growth of porous coordination polymers (PCPs) on 3D conductive substrate to synthesize porous electrocatalysts for water splitting.^16^ On the one hand, PCPs are a class of structurally ordered porous materials, which have been proven to be promising precursors for preparing efficient electrocatalysts.^17^ Owing to the large active specific surface area and the uniform incorporation of derived carbon, these PCP‐derived materials exhibit impressive electrochemical activity for both HER or OER.^18^ On the other hand, due to the tight connection to the conductive substrates of the directly grown PCPs,^19^ the PCP‐derived electrocatalysts can be closely and strongly immobilized on the current collector,^20^ which is beneficial for boosting the electron transportation, improving electrochemically active sites, and stabilizing the working electrodes. However, it is vital and a challenging work to develop simple and efficient approach to fabricate electrocatalysts with superior activities for driving large‐current‐density (>500 mA cm^−2^) OER at low overpotentials.

In this work, a facile and scalable method was explored to prepare hierarchical bifunctional electrocatalyst by directly growing bimetallic Co‐Fe Prussian blue analogue (PBA) on the NF, which was further phosphorized into 3D bifunctional porous Fe‐CoP electrocatalyst. The obtained Fe‐CoP/NF self‐supported electrode exhibits excellent electrocatalytic performance for the OER and could deliver large current densities of 500 and 1000 mA cm^−2^ at pretty low overpotentials of 295 and 428 mV, respectively. Moreover, the Fe‐CoP electrode can also function as a highly active electrocatalyst for HER with a low overpotential of 78 mV at 10 mA cm^−2^ current density in alkaline solution. Thus, the bifunctional electrocatalyst requires a cell voltage of only 1.49 V for the overall water splitting to achieve a 10 mA cm^−2^ current density and could sustain high catalytic performance for at least 50 h in 1.0 m KOH solution. We report for the first time, to the best of our knowledge, a catalyst that can both drive large‐current‐density oxygen evolution to meet the strict criteria of industrial applications and efficiently catalyze overall water splitting.

## 2 Results and Discussion

**Figure 1:**
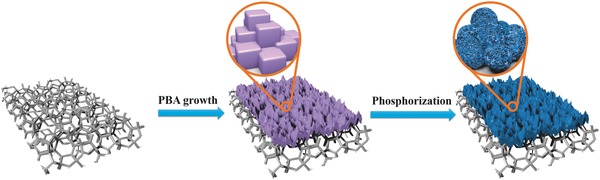
Schematic illustration of the preparation of porous Fe‐CoP/NF electrode.

**Figure 2:**
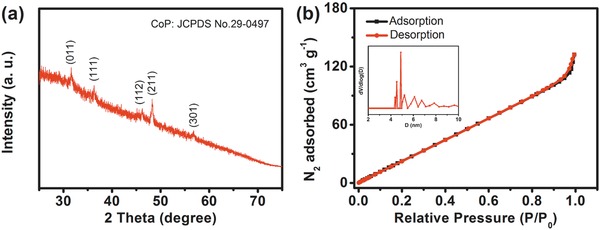
a) XRD pattern of Fe‐CoP scraped from the NF. b) N_2_ adsorption–desorption isotherms of porous Fe‐CoP (inset corresponding to pore size distribution of Fe‐CoP).

With super high electrical conductivity and 3D macroporous feature (Figure S1, Supporting Information), the low‐cost commercial NF was utilized as a supporting substrate for distributing the catalysts. As presented in **Figure** **1**, a piece of cleaned NF was immersed in a mixed solution of CoCl_2_.6H_2_O, sodium citrate, and K_3_[Fe(CN)_6_]. After 24 h stirring, the Co‐Fe PBA nanocubes were directly grown on NF through a gentle coprecipitation method (Figures S2 and S3, Supporting Information). Powder X‐ray diffraction (XRD) pattern of the as‐synthesized precursor (Co‐Fe PBA/NF) is presented in Figure S2 (Supporting Information). The diffraction peaks of Co‐Fe PBA matched well with the reported data,^21^ suggesting the successful preparation of pure KCo[Fe(CN)_6_]·3H_2_O on NF. As revealed by the scanning electron microscopy (SEM) (Figure S3, Supporting Information), the Co‐Fe PBA nanocubes are highly uniform with an average side length of 240 nm, and homogeneously cover the surface of NF and fabricated 3D macroporous structure. Then, the Fe‐CoP/NF electrode was obtained by thermal phosphorization of the Co‐Fe PBA/NF. After phosphorization, the morphology of Co‐Fe PBA nanocubes was converted to irregular nanospheres, which still maintained the 3D macroporous structure (Figure S4, Supporting Information).^22^ XRD measurement was utilized to analyze the phase and crystalline features of the as‐prepared Fe‐CoP. As shown in **Figure** **2**a, the diffraction peaks at 31.60°, 36.31°, 46.23°, 48.13°, and 56.78° can be assigned to the (011), (111), (112), (211), and (301) crystalline planes of CoP (JCPDS No. 29–0497), manifesting the existence of CoP in the Fe‐CoP.^23^

**Figure 3:**
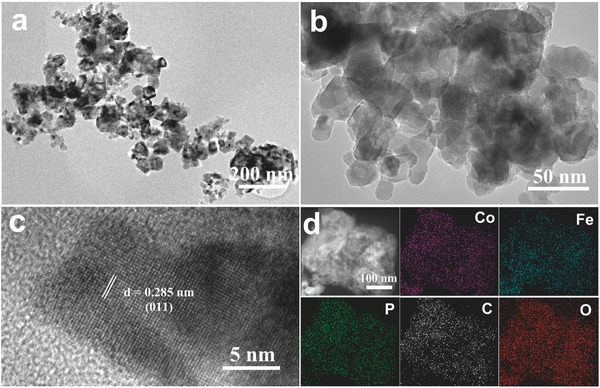
a,b) TEM and c) HRTEM images of Fe‐CoP. d) HAADF‐STEM image and EDS elemental mapping images for Fe‐CoP.

Transmission electron microscopy (TEM) analysis reveals that the nanosphere exhibits hierarchical nanoporous structure composed of small nanosheets (**Figure** **3**a,b). In order to further investigate the nanoporous structure, the nitrogen adsorption experiment was carried out to measure the specific surface area of Fe‐CoP. The result suggests that the nanoporous Fe‐CoP catalyst has a large Brunauer–Emmett–Teller specific surface area of 177 m^2^ g^−1^ with an average pore diameter of 4.3 nm (Figure 2b). The hierarchical‐pore architecture with the coexistence of mesopores and macropores not only affords abundant active sites, but also benefits to the contact of electrolyte and the release of gases, and improves mass transfer in their pores.[[qv: 17d,19,23]] As shown in Figure 3c, high‐resolution transmission electron microscopy (HRTEM) image clearly displays the crystalline nanoparticles embedded in amorphous carbon layers. These crystalline nanoparticles possess of apparent lattice fringes with lattice spacing of 0.285 nm, in accordance with the interplanar spacings of the (011) crystal plane of CoP. The result suggests the existence of crystalline CoP nanoparticles in Fe‐CoP,^24^ which was also confirmed by the XRD result. Scanning transmission electron microscopy (STEM) with high‐angle annular dark field (HAADF) and corresponding energy dispersive X‐ray spectroscopy (EDS) elemental mapping images demonstrate the presence and uniform distribution of Co, Fe, P, O, C elements (Figure 3d), indicating the iron element homogeneously distributed in the Fe‐CoP nanoparticles. Elemental dispersive X‐ray spectroscopy also confirms the existence of Co, Fe, P, O, and C elements in Fe‐CoP (Figure S5, Supporting Information). The inductively coupled plasma‐mass spectroscopy (ICP‐MS) analysis indicates that the actual atomic ratio of Co and Fe in the Fe‐CoP is ≈3:2.

**Figure 4:**
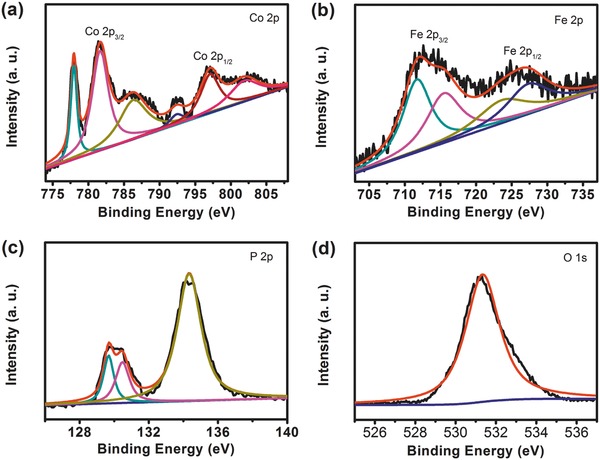
High resolution XPS spectra of a) Co 2p, b) Fe 2p, c) P 2p, and d) O 1s for Fe‐CoP.

Further, X‐ray photoelectron spectroscopy (XPS) was conducted to characterize the hierarchical Fe‐CoP nanoparticles. As shown in **Figure** **4**, in the high resolution Co 2p XPS spectrum, the peaks at 778.0 and 792.8 eV are assigned to the Co 2p_3/2_ and Co 2p_1/2_ binding energies of the reduced Co^0^ in Fe‐CoP. The peaks at 781.2 and 797.3 eV as well as two satellite peaks at 785.9 and 802.6 eV are attributed to the oxidized Co species (Co^2+/3+^) resulting from the surface oxidation of Fe‐CoP.^24^ The high resolution Fe 2p spectrum reveals two spin–orbit doublets. The first doublet at 711.5 and 723.6 eV corresponds to the binding energies of Fe 2p_3/2_ and Fe 2p_1/2_, indicating the presence of Fe^2+^ in Fe‐CoP.^25^ The second one at 715.6 and 727.3 eV can be assigned to Fe^3+^.^25^ The high resolution P 2p spectrum shows three main peaks at 129.6, 130.4, and 134.2 eV, respectively. The first two peaks are attributed to the metal phosphides, and the other peak at 134.2 eV corresponds to the oxidized metal‐phosphate species.[[qv: 12d]] It is worth noting that no peaks of FeP have been observed, indicating the fabrication of single‐phase Fe‐phosphate in the Fe‐CoP.[[qv: 14a,26]] For the O 1s XPS, the peak at 531.2 eV can be assigned to the P—O bond.[[qv: 12c]] The XPS study indicates that the surface compositions of Fe‐CoP are the CoP, Fe^2+^, Fe^3+^, and phosphate species.

Electrocatalytic performance of the as‐prepared Fe‐CoP/NF for OER was evaluated in a 1.0 m KOH solution with a scan rate of 1 mV s^−1^ in a three‐electrode system. For comparison, the Fe‐Co oxide (CoFe_2_O_4_, see Figure S6 in the Supporting Information) and commercial IrO_2_ supported on NF with the same loading mass (the mass loading of Fe‐CoP is 4.20 mg cm^−2^) were also investigated. An *iR* compensation was applied to all initial electrochemical data. As shown in Figure 4a, linear sweep voltammetry (LSV) curves reveal that the Fe‐CoP/NF self‐supported electrode presents the lowest overpotential of 190 mV at 10 mA cm^−2^ current density, which is much smaller than those of CoFe_2_O_4_/NF (265 mV) and IrO_2_/NF (286 mV). These results confirmed that the Fe‐doped CoP material could act as a highly efficient electrocatalyst for OER reaction under alkaline conditions (Table S1, Supporting Information). To further assess the intrinsic catalytic activity, the OER kinetics was measured by corresponding Tafel plots. As shown in Figure 4b, the Tafel slope of Fe‐CoP/NF is 36 mV dec^−1^, much lower than that of CoFe_2_O_4_/NF (62 mV dec^−1^) and IrO_2_/NF (90 mV dec^−1^), indicating a beneficial OER kinetics of Fe‐CoP/NF. The turnover frequency (TOF) value of Fe‐CoP for OER is 3.09 min^−1^ at an overpotential of 300 mV, which is much better than that of CoFe_2_O_4_ (0.27 min^−1^). In addition, the electrocatalytic experiments with Ni_2_P/NF substrate and the NF substrate were all performed, which indicate the catalytic activity of Fe‐CoP/NF electrode for OER mainly originated from the Fe‐doped CoP (Figure S7a, Supporting Information). Such a low overpotential and small Tafel slope sufficiently affirmed that the Fe‐CoP/NF electrode possesses a remarkable electrocatalytic activity for OER.

The electrochemical impedance spectroscopy (EIS) was performed to present the charge‐transfer resistance (*R*_CT_) of Fe‐CoP/NF and CoFe_2_O_4_/NF electrodes. As shown in Figure S8a (Supporting Information), the Fe‐CoP/NF electrode affords smaller *R*_CT_ value with the faster charge‐transfer ability, which illustrates the quick reaction kinetic of the Fe‐CoP/NF electrode. Further, the double‐layer capacitance (*C*_dl_), which is generally used to estimate the electrochemical surface area (ECSA), was measured to evaluate the catalytic performance of Fe‐CoP/NF electrode. As expected, the Fe‐CoP/NF electrode has a much higher *C*_dl_ value (19.43 mF cm^−2^) than that of the CoFe_2_O_4_ electrode (6.3 mF cm^−2^), revealing that the Fe‐CoP/NF electrode has more active sites for OER (Figure S9, Supporting Information). These experimental data demonstrate that the substantial increase of the conductivity and active sites greatly contribute to the high catalytic efficiency of Fe‐CoP for OER.

**Figure 5:**
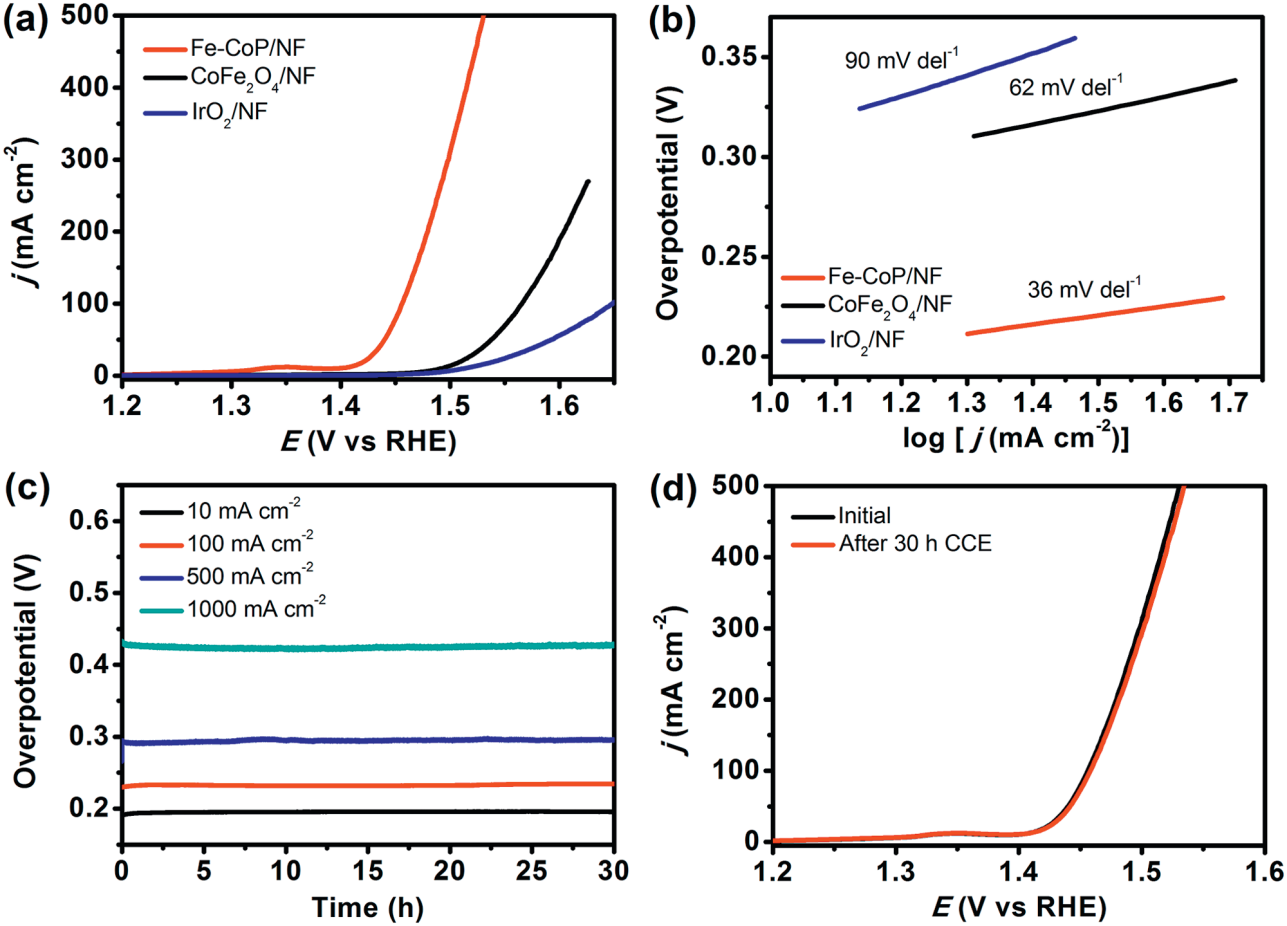
a) LSV curves and b) Tafel plots of Fe‐CoP/NF, CoFe_2_O_4_/NF, and IrO_2_/NF recorded at a scan rate of 1 mV s^−1^ in 1.0 m KOH. c) Current density traces of CCE at 10, 100, 500, and 1000 mA cm^−2^ of Fe‐CoP/NF in 1.0 m KOH solution. d) LSV curves of Fe‐CoP/NF before (black line) and after (red line) CCE for OER at 10 mA cm^−2^ for 30 h.

As well known, the electrocatalytic performance for the OER is imperative to be further enhanced for constructing more efficient catalyst working well at large current densities needed for industrial applications. Most of these promising nonprecious OER catalysts, however, appeared to operate inefficiently and unstably at large current densities.^15^ It is interesting to note that for the hierarchical‐pore Fe‐CoP/NF electrode, the large current densities of 100, 500, and even 1000 mA cm^−2^ were readily achieved at quite low overpotentials of 227, 295, and 428 mV, respectively. High durability of electrocatalyst is also of great significance for practical applications. In this paper, controlled‐current electrolysis (CCE) was used to determine the stability of Fe‐CoP for OER at the current density of 10 mA cm^−2^. As shown in **Figure** **5**c, there is no obvious change in the CCE process for at least 30 h, demonstrating an excellent stability of the Fe‐CoP/NF electrode for OER. Notably, the Fe‐CoP/NF electrode still maintained excellent stability at a high current density of 100, 500, and even 1000 mA cm^−2^ (Figure 5c). Moreover, the excellent stability of Fe‐CoP/NF electrode was also confirmed by the LSV measurements after 30 h CCE for OER at 10 mA cm^−2^, in which the LSV curves before and after CCE are almost identical (Figure 5d).

We further characterized the stability of the Fe‐CoP catalyst after OER by the SEM, TEM, and XPS. The SEM images show that the Fe‐CoP/NF electrode still maintained the 3D macroporous structure (Figure S10a, Supporting Information), and the TEM study reveals that the Fe‐CoP catalyst retained the nanosheet morphology (Figure S10b, Supporting Information). Moreover, XPS was performed to explore the composition of Fe‐CoP after OER. As shown in Figure S11 (Supporting Information), in the high resolution Co 2p XPS spectrum, the peaks at 780.3 and 795.6 eV are attributed to Co^3+^, suggesting the formation of CoOOH.^27^ The high resolution Fe 2p spectrum is displayed in Figure S11b (Supporting Information), indicating the presence of Fe^3+^.^28^ The bond energy of P 2p at 129.8 eV disappeared, which suggests the oxidation of P center on the surface of Fe‐CoP.^25^, ^29^ The XPS study confirms that the Fe‐CoP catalyst is partially oxidized to the Co(Fe) oxyhydroxide species, which is the active species to boost the OER.^29^, ^30^ As shown in Figure S10b (Supporting Information), HRTEM image shows the apparent lattice fringes with lattice spacing of 0.248 nm, which are consistent with the interplanar spacing of (111) crystal plane of CoP (JCPDS No. 29–0497).

**Figure 6:**
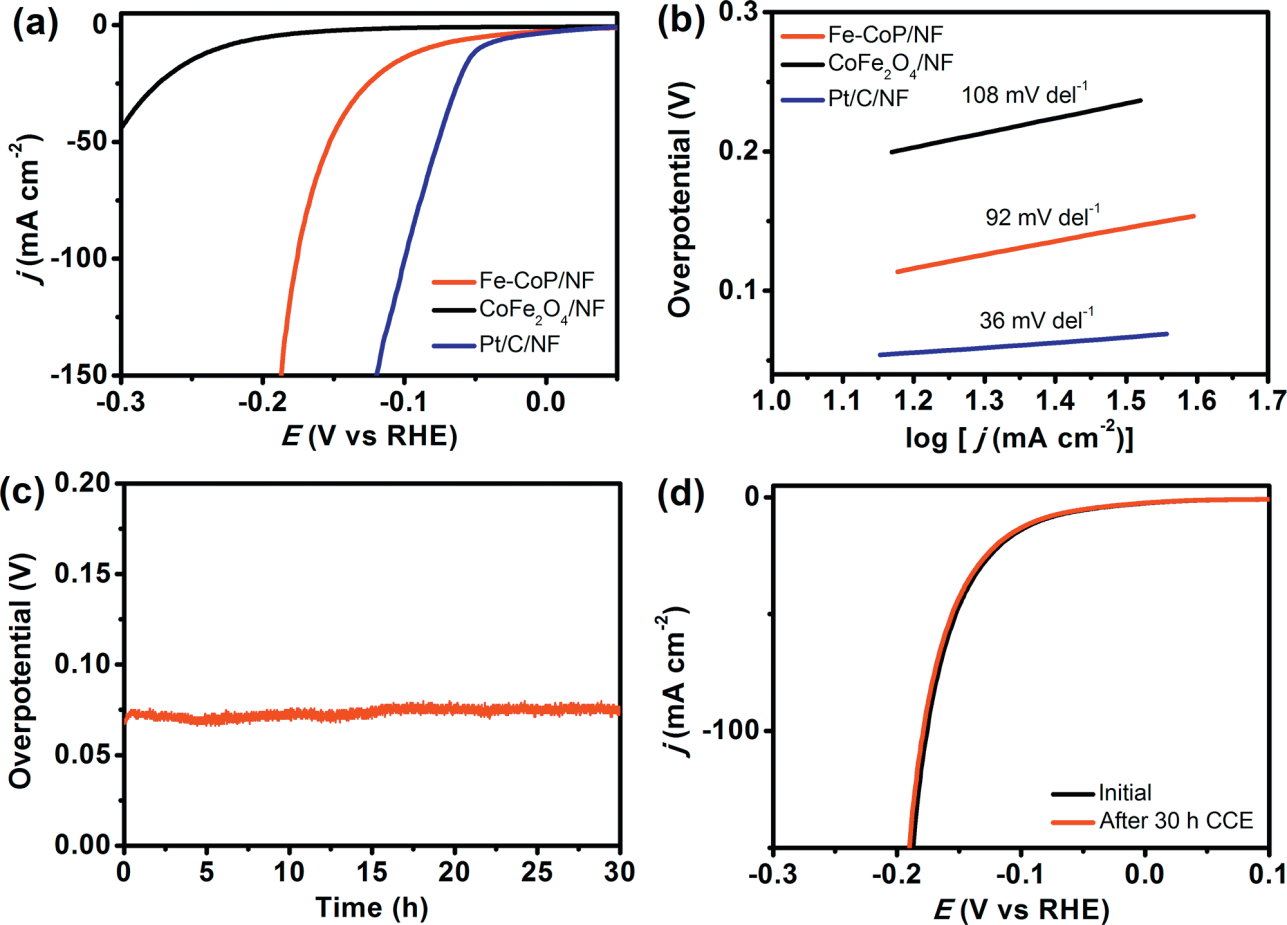
a) LSV curves and b) Tafel plots of Fe‐CoP/NF, CoFe_2_O_4_/NF, and Pt/C/NF recorded at a scan rate of 1 mV s^−1^ in 1.0 m KOH solution. c) Current density trace of CCE at 10 mA cm^−2^ of Fe‐CoP/NF in 1.0 m KOH. d) LSV curves of Fe‐CoP/NF before (black line) and after (red line) CCE for HER at 10 mA cm^−2^ for 30 h.

The electrocatalytic HER with Fe‐CoP/NF electrode was also assessed in 1.0 m KOH solution using a typical three‐electrode cell. The LSV curves of these electrodes obtained at a scan rate of 1 mV s^−1^ after *iR* compensation are shown in **Figure** **6**a. It is worth mentioning that the Fe‐CoP/NF hierarchical‐pore electrode exhibits a relatively low overpotential of 78 mV at 10 mA cm^−2^ current density, which is comparable to many reported nonprecious HER catalysts (Table S2, Supporting Information), and much lower than that of CoFe_2_O_4_/NF (242 mV). Further, the LSV curves of Ni_2_P/NF substrate and NF were also performed, illustrating that the efficient HER performance mostly originates from the Fe‐CoP, rather than the Ni_2_P/NF substrate or the NF substrate (Figure S7b, Supporting Information). As shown in Figure 6b, the Fe‐CoP/NF electrode delivers a low Tafel slope of 92 mV dec^−1^, superior to that of CoFe_2_O_4_ (108 mV dec^−1^), illustrating a rapid HER rate. The TOF value of Fe‐CoP for HER is 4.14 min^−1^ at an overpotential of 200 mV, much superior to that of CoFe_2_O_4_ (0.252 min^−1^). The low overpotential and the small Tafel slope demonstrate that the Fe‐CoP/NF electrode possesses the efficient electrocatalytic performance for the HER. The EIS measurements were carried out to detect the charge‐transfer resistance of these catalysts. As shown in Figure S8b (Supporting Information), the calculated *R*_CT_ value (3.2 ohm) of the Fe‐CoP/NF electrode is much less than that of CoFe_2_O_4_/NF electrode, indicating the fast charge‐transfer ability of the Fe‐CoP/NF electrode. CCE curve is depicted as Figure 6c, where Fe‐CoP/NF maintained stable HER catalytic performance for at least 30 h at the current density of 10 mA cm^−2^. Furthermore, the LSV curves before and after CCE for HER (Figure 6d) also confirmed the high durability of Fe‐CoP in 1 m KOH solution. The stability the Fe‐CoP catalyst after HER was further studied by the SEM, TEM, and XPS. The SEM and TEM images reveal that the Fe‐CoP catalyst retained the 3D macroporous structure and the nanosheet morphology (Figure S12, Supporting Information). In the HRTEM image, the lattice spacing of 0.190 nm (Figure S12b, inset, Supporting Information) in accordance with the lattice fringes of CoP of (211) crystal plane (JCPDS No. 29–0497) was clearly detected. The XPS result of Fe‐CoP catalyst after HER was almost identical to that of the fresh Fe‐CoP catalyst (Figure S13, Supporting Information), demonstrating that the chemical composition of Fe‐CoP remains the same after the 30 h electrolysis. The above results sufficiently reveal the high stability of Fe‐CoP for HER in 1 m KOH aqueous solution. Besides, control experiments demonstrate that the existence of the Fe element plays a key role in boosting the OER and HER catalytic activity of the electrocatalyst. Also shown in Figure S14 (Supporting Information), the Co‐Co and Fe‐Fe PBAs were all synthesized and phosphorized under the same conditions (abbreviated as Co‐P and Fe‐P). The as‐obtained samples Co‐P and Fe‐P were loaded on NF with the same loading mass of Fe‐CoP for comparison. As shown in Figure S15 (Supporting Information), Fe‐CoP exhibits the best catalytic performance for both OER and HER among these three examples, illustrating that the incorporation of Fe elements into the hierarchical‐pore Fe‐CoP/NF electrode could dramatically enhance its catalytic activity.[[qv: 12d,24]] Also, the bulk Fe‐CoP/NF electrode was prepared by loading the same mass of Fe‐CoP (4.20 mg cm^−2^) on NF. The CCE experiments show that the Fe‐CoP/NF electrode exhibits much better electrocatalytic performance for both OER and HER than that of the bulk Fe‐CoP/NF electrode (Figure S16, Supporting Information).

**Figure 7:**
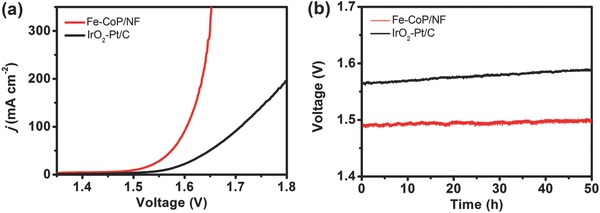
a) LSV curves of Fe‐CoP/NF (red) as bifunctional catalyst in 1.0 m KOH solution for overall water splitting. IrO_2_ and Pt/C as OER and HER benchmarks were measured for comparison (black). b) Current density traces of CCE at 10 mA cm^−2^ for overall water splitting in 1.0 m KOH solution.

Inspired by the impressive electrocatalytic performance of Fe‐CoP/NF for both OER and HER, the hierarchical‐pore Fe‐CoP/NF electrodes were used as both anode and cathode to fabricate an electrolyzer for overall water splitting in 1.0 m KOH aqueous solution. Meanwhile, a noble‐metal‐based electrolyzer, constructed by commercial benchmarks of IrO_2_ and Pt/C on NF, was utilized to perform the control experiments. As shown in **Figure** **7**a, the LSV curves indicate that the Fe‐CoP‐based electrolyzer merely demands the cell voltage of 1.49 V to afford 10 mA cm^−2^ current density for overall water splitting. This performance is among the best reported values for bifunctional electrocatalysts, and much better than that of the IrO_2_‐Pt/C‐based electrolyzer (1.58 V) (Table S2, Supporting Information). Besides the high catalytic efficiency, the Fe‐CoP‐based electrolyzer exhibits remarkable stability during a 50 h CCE at 10 mA cm^−2^ (Figure 7b). Meanwhile, no noticeable difference was observed in the LSV curves before and after 50 h CCE, which further confirm its high stability (Figure S17, Supporting Information). The actual H_2_ and O_2_ yields matched well with the theoretical values, suggesting that the Faradaic efficiency of Fe‐CoP was nearly 100% (Figure S18, Supporting Information).

## 3 Conclusion

In summary, we have developed a simple and practical approach to fabricate the hierarchical‐pore bifuntional electrocatalyst for large‐current‐density OER and overall water splitting by phosphorization of CP/NF, obtained by directly growing bimetallic Co‐Fe PBA on the 3D porous conductive NF substrate. The as‐prepared Fe‐CoP/NF electrode exhibits efficient electrocatalytic efficiency and high stability for both OER and HER, requiring only the cell voltage of 1.49 V to afford 10 mA cm^−2^ current density in 1.0 m KOH solution, which is much better than that of the IrO_2_‐Pt/C‐based electrolyzer. The hierarchical‐pore architecture with the coexistence of mesopores and macropores could afford abundant active sites and benefits to the contact of electrolyte and the release of gases of the catalysts. Thus, this catalyst could deliver large current densities of 500 and 1000 mA cm^−2^ at low overpotentials of 295 and 428 mV, respectively, to meet the strict criteria of industrial applications. This work not only provides a facile and efficient strategy for the development of effective and low‐cost electrocatalysts for overall water splitting, but also supplies an efficient material design method for exploring hierarchical‐pore electrode to deliver the large‐current‐density (>500 mA cm^−2^) OER at low overpotentials to meet the demand for industrial applications.

## 4 Experimental Section

*Synthesis of Co‐Fe PBA Nanocubes on NF (Co‐Fe PBA/NF)*: NF was cleaned by sonication in acetone, ethanol, and a HCl aqueous solution (3.0 m) for 15 min, respectively. At first, CoCl_2_.6H_2_O (143 mg) and sodium citrate (397 mg) were dissolved in 40 mL distilled water with stirring. Next, 132 mg K_3_[Fe(CN)_6_] was added, and the mixture was stirred for another 5 min to achieve a clear solution. Then, the obtained clear solution was transferred into a 50 mL beaker, and a piece of cleaned NF (2 cm × 4 cm) was immersed into the solution for 24 h without interruption. Finally, the obtained Co‐Fe PBA/NF was washed with ethanol and dried at 50 °C.

The isolated Co‐Fe PBA was synthesized in the absence of NF under the similar method. To prepare the Co‐Co PBA and Fe‐Fe PBA, K_3_[Co(CN)_6_] (132 mg) and FeSO_4_.7H_2_O (167 mg) were used to replace K_3_[Fe(CN)_6_] and CoCl_2_.6H_2_O, respectively, the synthetic procedure was similar to that for Co‐Fe PBA.

*Synthesis of Fe‐CoP/NF*: 1.0 g of NaH_2_PO_2_ was placed at the upstream side of a tube furnace, and a piece of Co‐Fe PBA/NF (2 cm × 4 cm) was located at the downstream side of the furnace. Then, the furnace was heated to 400 °C with a heating rate of 2 °C min^−1^ under N_2_ flow, and maintained at 400 °C for 3 h. The Fe‐CoP/NF was obtained after naturally cooling to room temperature. To prepare the Fe‐CoP, Co‐P, and Fe‐P, the Co‐Fe PBA, Co‐Co PBA, and Fe‐Fe PB were used as the precursors, respectively.

*Synthesis of CoFe_2_O_4_/NF*: To prepare CoFe_2_O_4_, a piece of Co‐Fe PBA/NF (2 cm × 4 cm) was located at the furnace. Then, the furnace was heated to 400 °C with a heating rate of 2 °C min^−1^ under N_2_ flow, and maintained at 400 °C for 3 h. The CoFe_2_O_4_/NF was obtained after naturally cooling down to room temperature.

*Characterization*: The morphology of catalysts was characterized by SEM (Hitachi SU8010) and TEM images (JEM2100F). Powder XRD data were obtained on a D8 ADVANCE X‐ray Diffractometer. XPS measurements were carried out on an ESCA Lab250 X‐ray photoelectron spectrometer. Nitrogen sorption isotherms were measured at 77 K using an automatic volumetric sorption apparatus (Micromertics ASAP 2020M). The exact contents of Co and Fe were analyzed by ICP‐MS.

*Electrochemical Measurements*: Electrocatalytic measurements were carried out by a CHI760E electrochemical workstation using a standard three‐electrode system with an Ag/AgCl (in 3 m KCl solution) reference electrode and a platinum counter electrode. For HER, a carbon rod was utilized as the counter electrode. The loading mass of Fe‐CoP catalyst on the NF is 4.20 mg cm^−2^. Therefore, the same amount of commercial IrO_2_ and Pt/C were loaded on NF for comparison. In a typical preparation procedure, catalysts (20 mg) were dispersed in 950 µL EtOH and 50 µL of 5% Nafion solution with sonication to obtain the homogeneous catalyst ink. Then, 210 µL of the ink was spread onto a piece of pretreated NF (1 cm^2^), and dried at room temperature. To prepare the bulk Fe‐CoP/NF, Co‐P/NF, and Fe‐P/NF electrodes, catalysts (20 mg) were dispersed in 950 µL EtOH and 50 µL of 5% Nafion solution with sonication to obtain the homogeneous catalyst ink. Then, 210 µL of the ink was spread onto a piece of pretreated NF (1 cm^2^), and dried at room temperature.

The Fe‐CoP/NF and CoFe_2_O_4_/NF self‐supported electrodes were directly used as the working electrodes. Before recording the electrochemical data, all the working electrodes were activated to a stable state by cyclic voltammetry (CV) at a scan rate of 100 mV s^−1^ in 1.0 m KOH solution with the sweep range of 1.0–1.6 V versus reversible hydrogen electrode (RHE) for OER (or 0–0.4 V vs RHE for HER). The LSV data were obtained at a scan rate of 1 mV s^−1^. The electrochemical double layer capacitance (*C*_dl_) was measured by CV measurements to determine the ECSA of the electrodes. The CV measurements were carried out in non‐Faradaic region (0.87–0.97 V vs RHE) at the sweep rates of 10, 20, 30, 40, and 50 mV s^−1^. A linear plot was acquired by plotting the measured capacitive currents at 0.92 V versus RHE against the scan rate. The linear slope is twice that of the *C*_dl_. EIS was performed with a frequency ranging from 0.01 Hz to 100 kHz. To determine the Faraday efficiency, two pieces of Fe‐CoP/NF (1 cm^2^) electrodes were utilized as the anode and cathode. The CCE was carried out at the 10 mA cm^−2^ current density. The yielding H_2_ and O_2_ were analyzed on a gas chromatography (Agilent 7820A‐GC, molecular sieve columns, thermal‐conductivity detector). The TOF values were calculated using the following equation: TOF = *jA*/(4 × *F* × *n*) for OER and TOF = *jA*/(2 × *F* × *n*) for HER, where *j* is the current density (mA cm^−2^), *A* is the area of the electrode, *F* is the faraday constant (96 485 C mol^−1^), and *n* is the number of moles of catalysts.

## Conflict of Interest

The authors declare no conflict of interest.

## Supporting information

Supplementary
