# Supplementary material for: Fe‐CoP Electrocatalyst Derived from a Bimetallic Prussian Blue Analogue for Large‐Current‐Density Oxygen Evolution and Overall Water Splitting
Source: Adv Sci (Weinh). 2018 Aug 14;5(10):1800949. doi: 10.1002/advs.201800949 (PMC6193147; doi:10.1002/advs.201800949)
Supplement: Supplementary file 1 — Supplementary [file ADVS-5-1800949-s001.pdf]

Copyright WILEY-VCH Verlag GmbH & Co. KGaA, 69469 Weinheim, Germany, 2018.

Supporting Information

for *Adv. Sci.,* DOI: 10.1002/advs.201800949

Fe-CoP Electrocatalyst Derived from a Bimetallic Prussian Blue Analogue for Large-Current-Density Oxygen Evolution and Overall Water Splitting

Li-Ming Cao, Yu-Wen Hu, Shang-Feng Tang, Andrey Iljin, Jia-Wei Wang, Zhi-Ming Zhang, and Tong-Bu Lu**

Supporting Information

Fe-CoP Electrocatalyst Derived from Bimetallic Prussian Blue Analogue for Large-Current-Density Oxygen Evolution and Overall Water Splitting

Li-Ming Cao, Yu-Wen Hu, Shang-Feng Tang, Andrey Iljin, Jia-Wei Wang, Zhi-Ming Zhang, and Tong-Bu Lu**

Li-Ming Cao, Yu-Wen Hu, Jia-Wei Wang, Prof. Tong-Bu Lu

MOE Key Laboratory of Bioinorganic and Synthetic Chemistry, School of Chemistry, Sun Yat-Sen University, Guangzhou 510275, China

E-mail: lutongbu@mail.sysu.edu.cn

Shang-Feng Tang, Prof. Zhi-Ming Zhang, Prof. Tong-Bu Lu

Institute for New Energy Materials & Low Carbon Technologies, School of Materials Science & Engineering, Tianjin University of Technology, Tianjin 300384, China

E-mail: zmzhang@email.tjut.edu.cn

Andrey Iljin

Department of Physics of Crystals, Institute of Physics, National Academy of Sciences of Ukraine, Prospect Nauki 46, Kyiv 03028 Ukraine

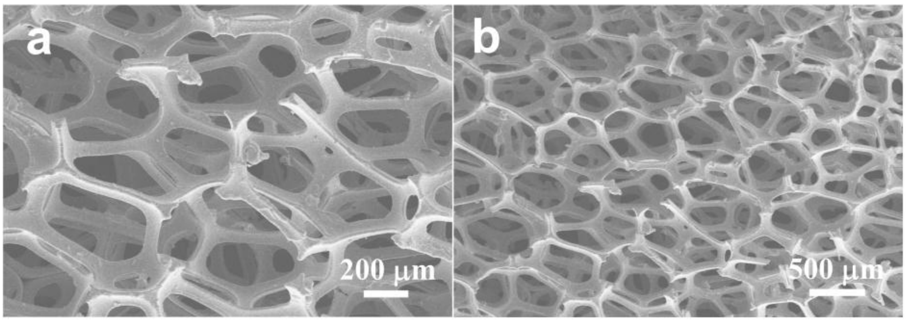

Figure S1. SEM images of NF.

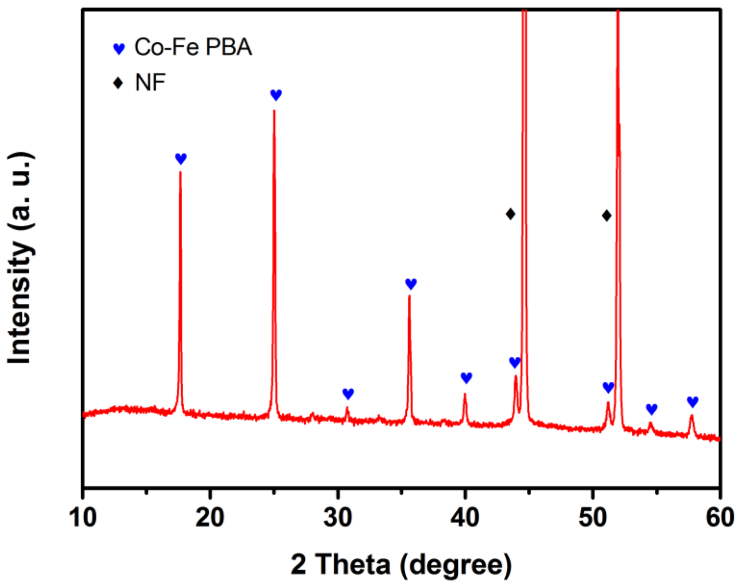

Figure S2. XRD pattern of Co-Fe PBA/NF.

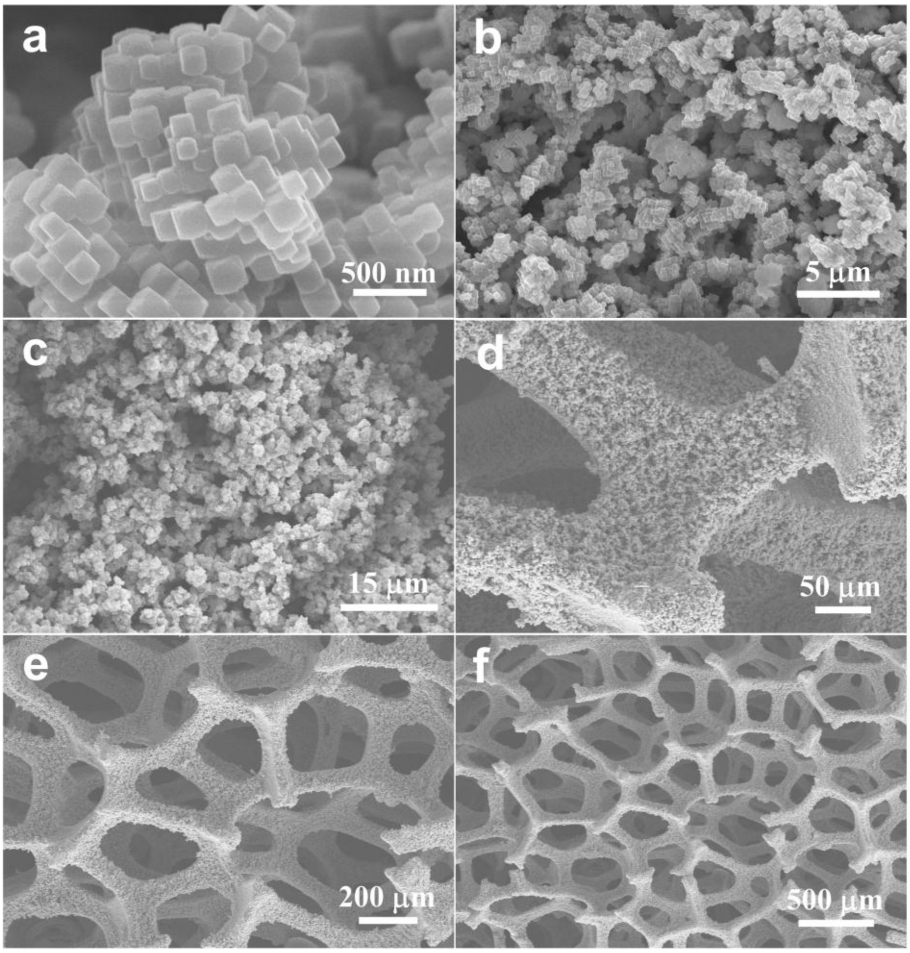

Figure S3. SEM images of Co-Fe PBA/NF.

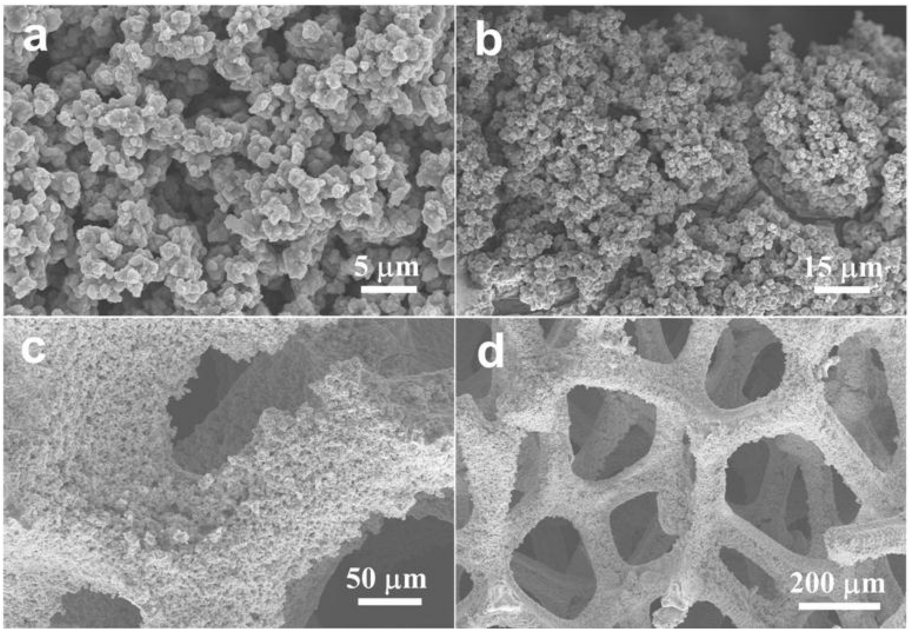

Figure S4. SEM images of Fe-CoP/NF.

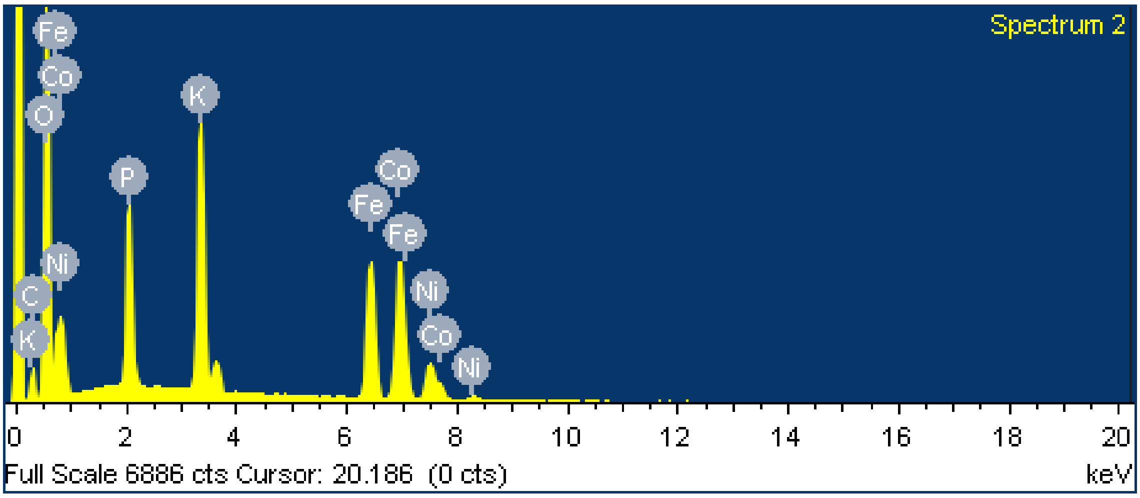

Figure S5. EDX spectrum of Fe-CoP/NF.

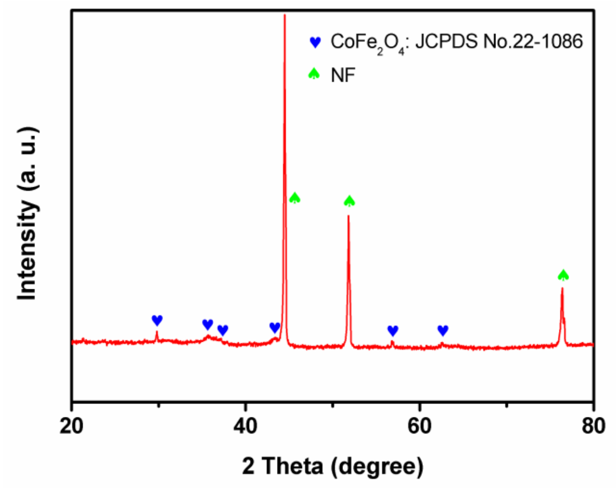

Figure S6. XRD pattern of $CoFe_2O_4$/NF.

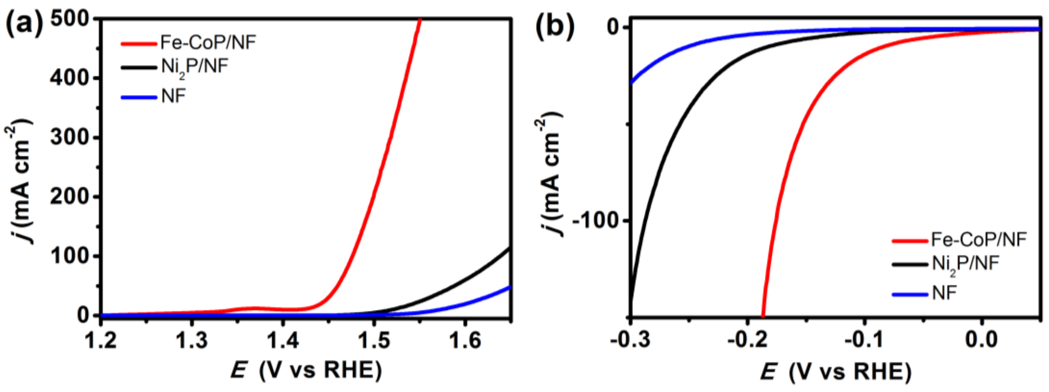

Figure S7. LSV curves of Fe-CoP/NF, Ni_2P/NF and bare NF recorded at a scan rate of 1 mV s^{-1} in 1.0 M KOH for (a) OER and (b) HER.

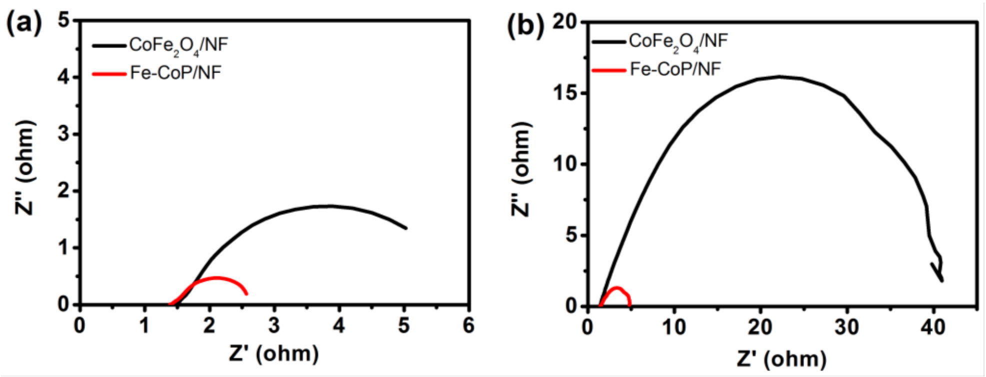

Figure S8. EIS plots of Fe-CoP/NF, $CoFe_2O_4$/NF measured at (a) the overpotential of 210 mV for OER and (b) the overpotential of 100 mV for HER.

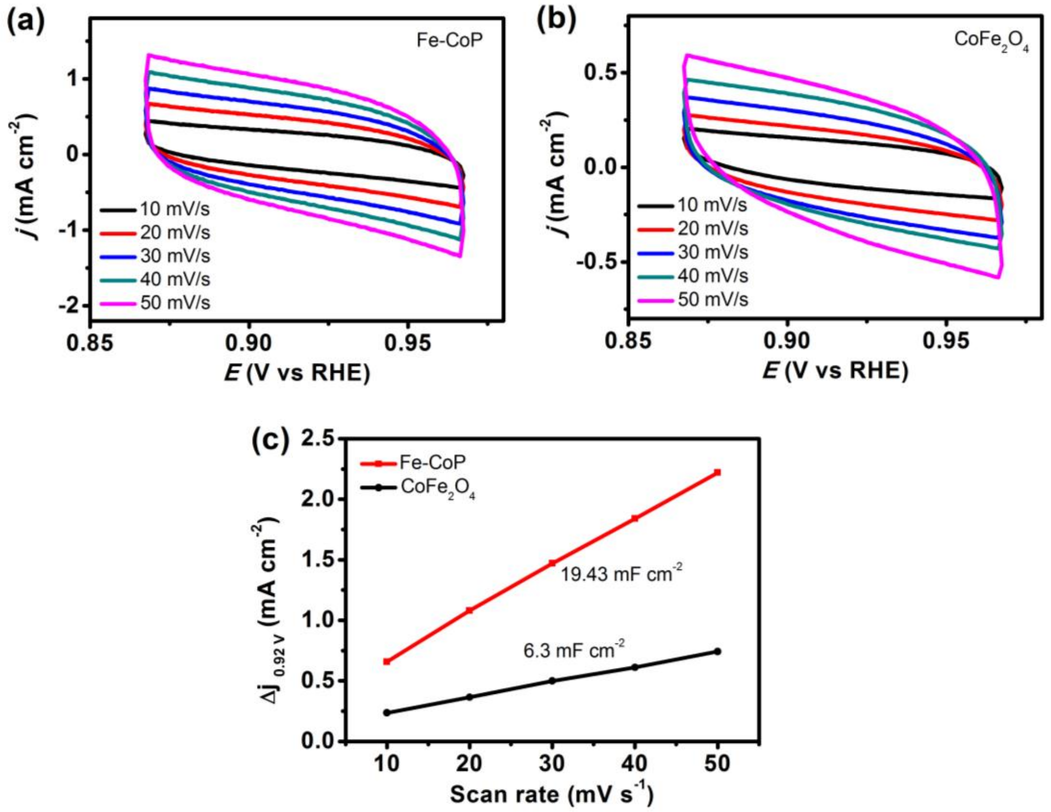

Figure S9. CVs of (a) Fe-CoP/NF, (b) $CoFe_2O_4$/NF in 1.0 M KOH solution at different scan rates, (e) the capacitive current density△$j_{0.92\ V}$ as a function of scan rate in the range of 0.87~0.97 vs. RHE.

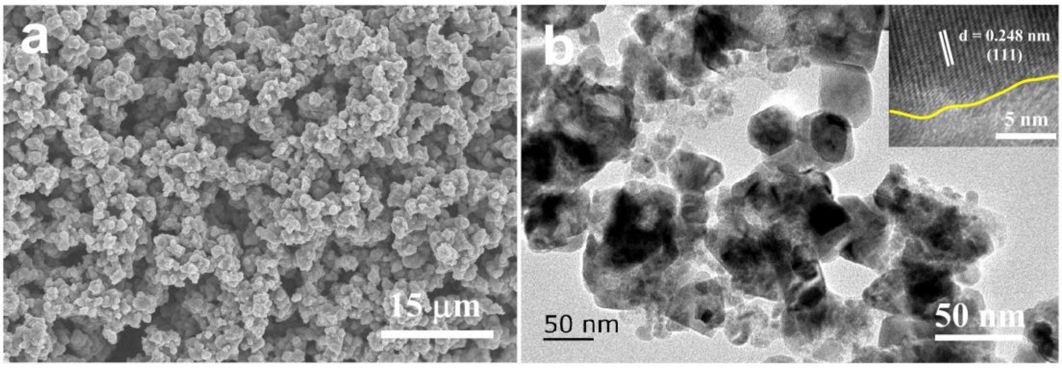

Figure S10. (a) SEM image of Fe-CoP after OER. (b) TEM image of Fe-CoP after OER, (inset) the HRTEM image of Fe-CoP after OER.

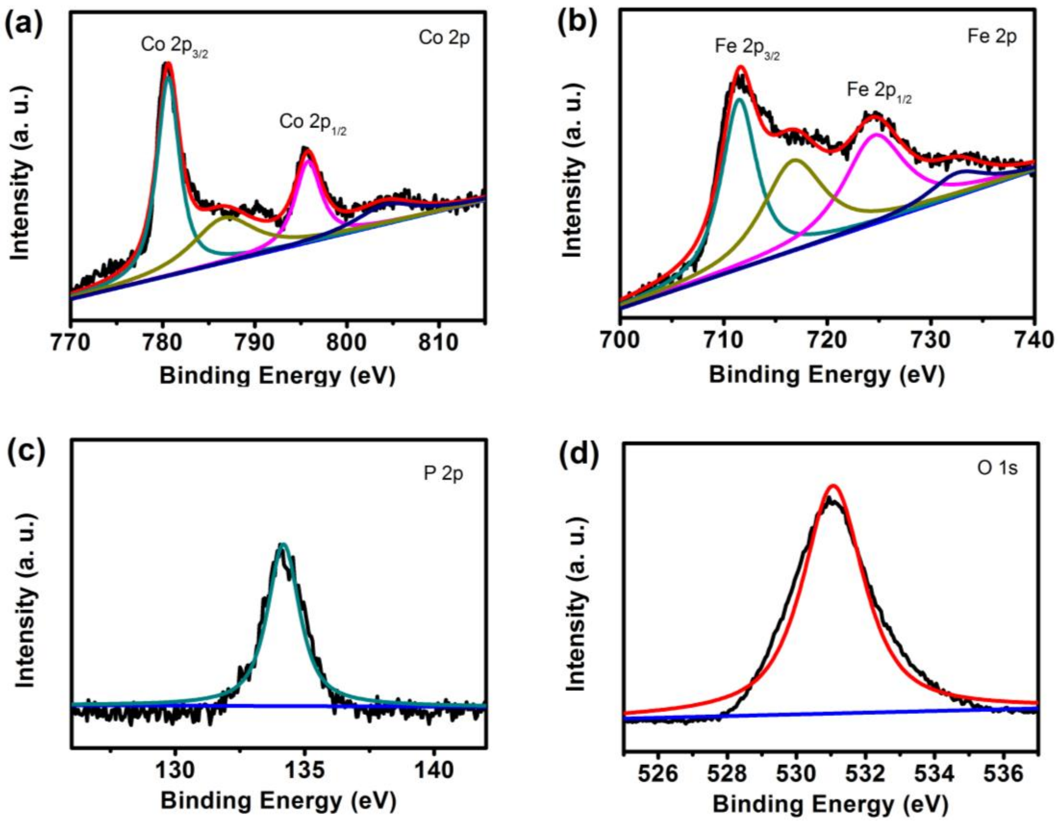

Figure S11. High resolution XPS spectra of (a) Co 2p, (b) Fe 2p, (c) P 2p, and (d) O 1s for Fe-CoP after OER.

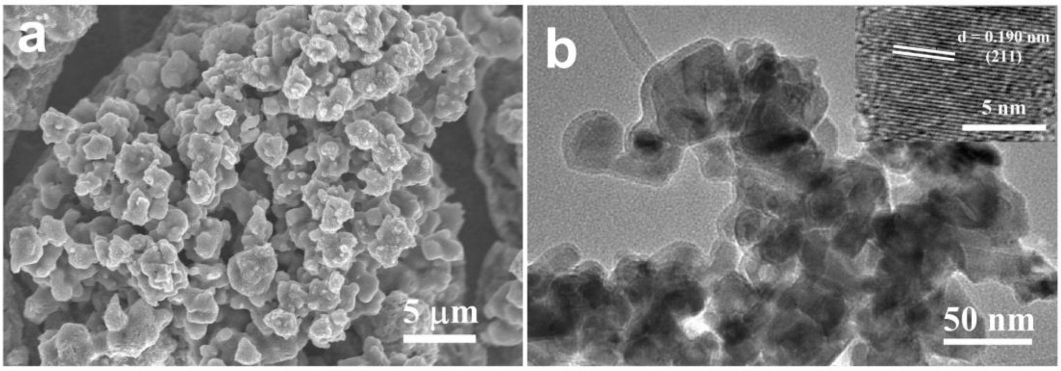

Figure S12. (a) SEM image of Fe-CoP after HER. (b) TEM image of Fe-CoP after HER, (inset) the HRTEM image of Fe-CoP after HER.

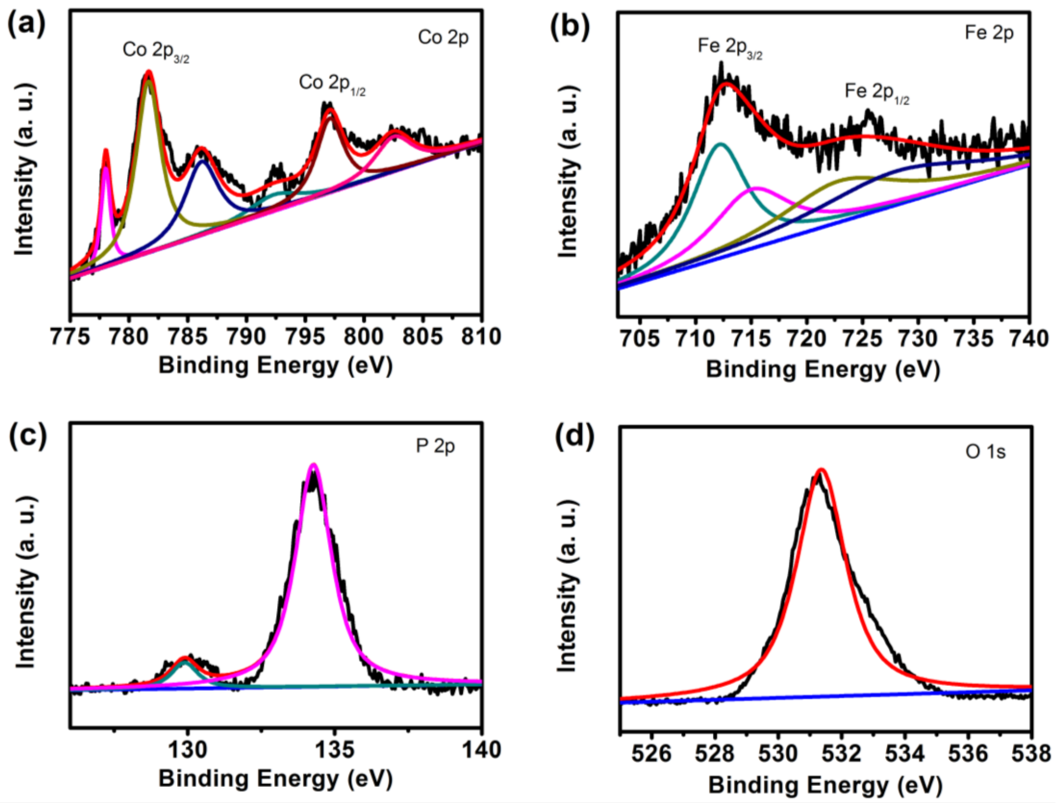

Figure S13. High resolution XPS spectra of (a) Co 2p, (b) Fe 2p, (c) P 2p, and (d) O 1s for Fe-CoP after HER.

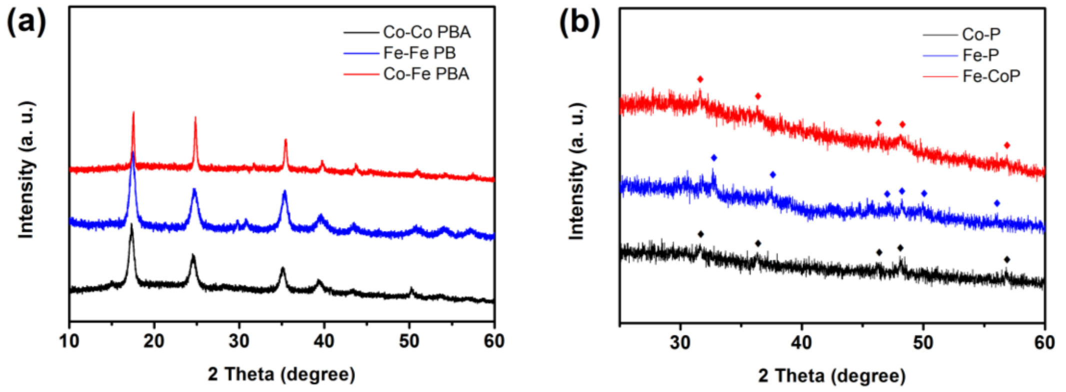

Figure S14. (a) XRD patterns of Co-Co, Fe-Fe and Co-Fe PBAs. (b) XRD patterns of Co-P, Fe-P and Fe-CoP.

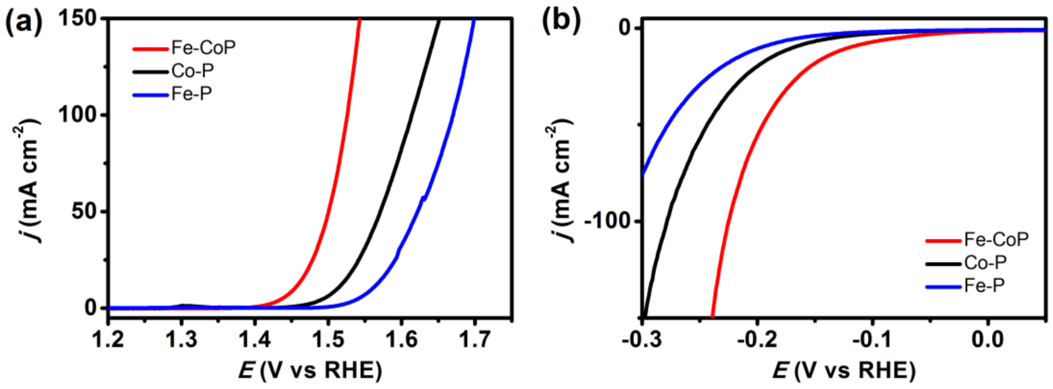

Figure S15. LSV curves of Fe-CoP, Co-P, and Fe-P powder samples recorded at a scan rate of 1 mV s^{-1} in 1.0 M KOH for (a) OER and (b) HER.

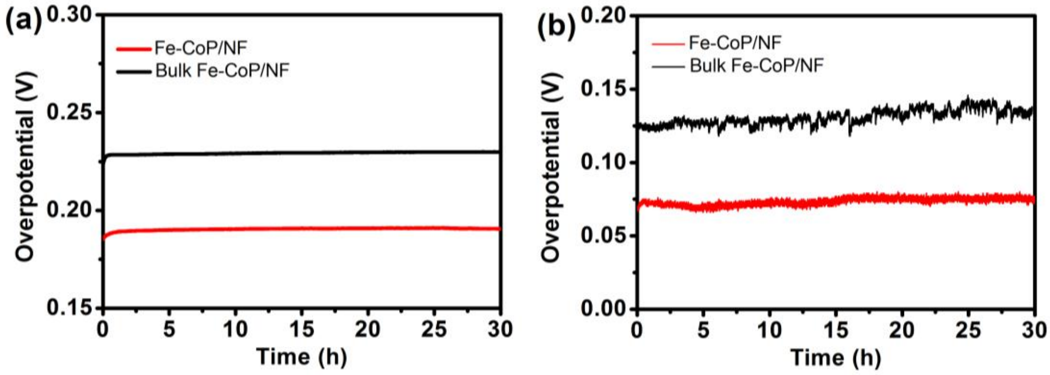

Figure S16. Current density trace of CCE at 10 mA cm^{-2} of Fe-CoP/NF and bulk Fe-CoP/NF in 1.0 M KOH for (a) OER and (b) HER.

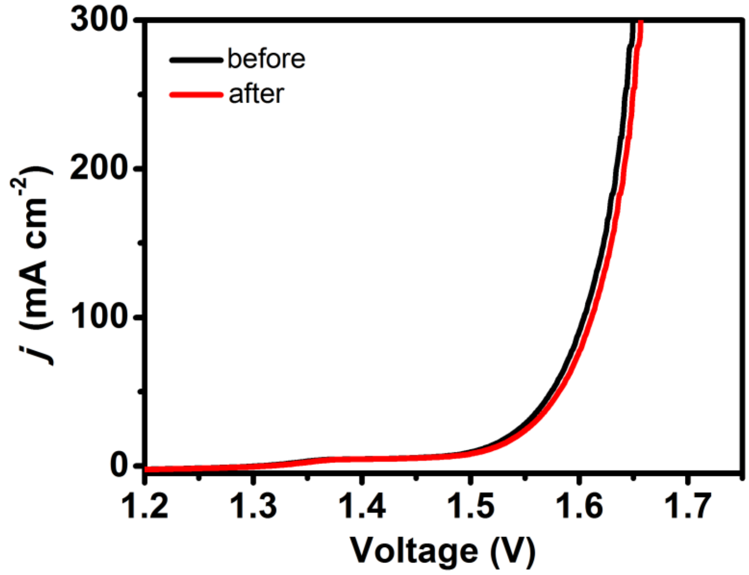

Figure S17. LSV curves of Fe-CoP/NF before (black line) and after (red line) 50 h of overall water splitting at 10 mA cm^{-2}.

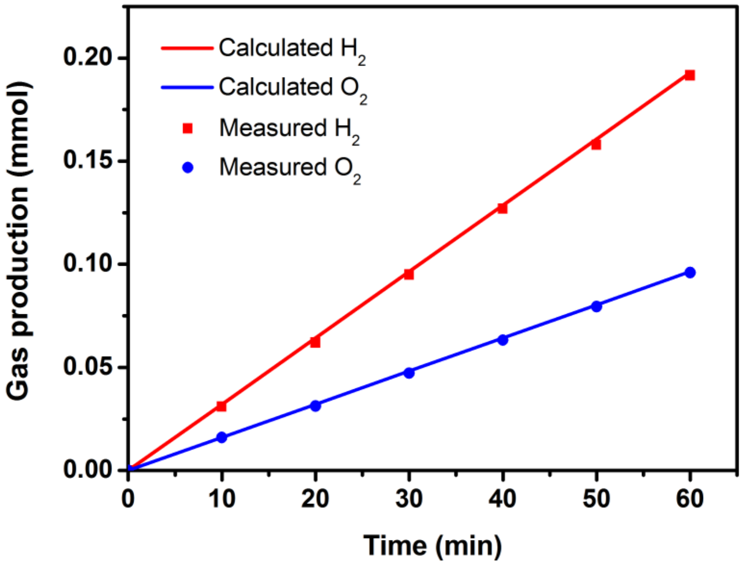

Figure S18. The measured and theoretical yields of O_2 and H_2 over time during electrolysis of Fe-CoP/NF at the current density of 10 mA cm^{-2}.

Table S1. Comparison of OER activity for some reported electrocatalysts.[a]

Catalyst	η at 10 mA cm^{-2} (mV)	η at 100 mA cm^{-2} (mV)	Tafel slope (mV del^{-1})	Substrate[b]	Reference
Fe-CoP	190	227	36	NF	This work
NiFeSe	N.A.	270	47.2	NF	*ACS Appl. Mater. Interfaces* **2016**, *8*, 19386-19392
$NiFeO_x$	230	260	31.5	CFP	*Nat. Commun.* **2015**, *6*, 7261
$Fe(PO_3)_2/Ni_2P$	177	221	51.9	NF	*Proc. Natl. Acad. Sci. U. S. A.* **2017**, *114*, 5607
Fe-doped Ni_3S_2	N.A.	253	65.5	NF	*J. Mater. Chem. A* **2015**, *3*, 23207-23212
Gelled FeCoW	190	250	N.A.	Au@NF	*Science* **2016,** *352*, 333
MoS_2/Ni_3S_2	218	290	88	NF	*Angew. Chem. Int. Ed.* **2016**, *55*, 6702-6707
$CoFe_2O_4$/C NRAs	240	290	45	NF	*Adv. Mater.* **2017,** *29,* 1604437
Co-Fe-P	244	N.A.	58	NF	*ACS Appl. Mater. Interfaces* **2017**, *9*, 362-

					370
NiCoP	242	330	64.2	CC	*ACS Catal.* **2017**, *7*, 4131-4137
$Ni_{1.5}Fe_{0.5}P$	264	293	55	CFP	*Nano Energy* **2017**, *34*, 472-480
$Ni_2P@FePO_x$	203	230	32	NF	*Chem. Sci.* **2018**, *9*, 1375-1384
$Fe\text{-}Ni_3S_2$	214	249	42	NF	*ACS Catal.* **2018**, *8*, 5431-5441
Fe-NiO	206	268	49.4	NF	*J. Catal.* **2018**, *358*, 243-252
$W_{0.5}Co_{0.4}Fe_{0.1}$	250	310	32	NF	*Angew. Chem. Int. Ed.* **2017**, *56*, 4502-4506
CoMnCH	N.A.	349	N.A.	NF	*J. Am. Chem. Soc.* **2017**, *139*, 8320-8328
Cu@NiFe LDH	199	281	27.8	CF	*Energy Environ. Sci.* **2017**, *10*, 1820-1827

[a] The electrolyte is 1.0 M KOH unless otherwise stated. η is the overpotential.

[b] NF = nickel foam; CFP = carbon fiber paper; CC = carbon cloth; CF = copper foam.

Table S2. Comparison of the overall water splitting activity for some reported bifunctional electrocatalysts.

Catalyst	OER η at 10 mA cm^{-2} (mV)	HER η at 10 mA cm^{-2} (mV)	Cell votage (10 mA cm^{-2} for overall water splitting) (mV)	Substrate	Reference
Fe-CoP	190	78	1.49	NF	This work
Co-P films	345	94	1.64	Cu foil	*Angew. Chem. Int. Ed.* **2015**, *54*, 6251-6254
$NiCo_2S_4$	260	210	1.63	NF	*Adv. Funct. Mater.* **2016**, *26*, 4661-4672
CoFePO	274	87	1.56	NF	*ACS Nano* **2016**, *10*, 8738-8745
NiCoP	280	32	1.58	NF	*Nano Lett.* **2016**, *16*, 7718-7725
MoS_2/Ni_3S_2	218	110	1.56	NF	*Angew. Chem. Int. Ed.* **2016**, *55*, 6702-6707
Porous MoO_2	260	25	1.53	NF	*Adv. Mater.* **2016**, *28*, 3785-3790
Ni-Fe-P	217	79	1.52	NF	*J. Mater. Chem. A* **2017**, *5*, 2496-2503
Co-Fe-P	244	295	1.60	NF	*ACS Appl. Mater. Interfaces* **2017**, *9*, 362-370

$Ni_{1.5}Fe_{0.5}P$	264	282	1.59	CFP	*Nano Energy* **2017**, *34*, 472-480
NiCoP	242	62	1.52	CC	*ACS Catal.* **2017**, *7*, 4131-4137
$Ni_2P@FePO_x$	205	75	1.51	NF	*Chem. Sci.* **2018**, *9*, 1375-1384
Fe-NiO	206	88	1.579	NF	*J. Catal.* **2018**, *358*, 243-252
Fe-Ni_3S_2	214	47	1.54	NF	*ACS Catal.* **2018**, *8*, 5431-5441
CoFe	220	110	1.64	NF	*Small* **2018**, *14*, 1702568
FeCoOOH	211	126	1.62	NF	*Chem. Eur. J.* **2018**, *24*, 4724-4728
Ni_3N-NiMoN	277	31	1.54	CC	*Nano Energy* **2018**, *44*, 353-363
O-CoMoS	272	97	1.60	CFC	*ACS Catal.* **2018**, *8*, 4612-4621
S-$NiFe_2O_4$	267	138	1.65	NF	*Nano Energy* **2017**, *40*, 264-273
$FePO_4$	218	123	1.54	NF	*Adv. Mater.* **2017**, *29*, 1704574
Cu@NiFe LDH	199	116	1.54	CF	*Energy Environ. Sci.* **2017**, *10*, 1820-1827
FeP/Ni_2P	154	14	1.42	NF	*Nat. Commun.* **2018**, *9*, 2551
Pt-CoS_2	300	24	1.54	CC	*Adv. Energy Mater.* **2018**, DOI: 10.1002/aenm.201800935
NiCoFe phosphate	240	231	1.52	NF	*Nanoscale* **2018**,*10*, 12975
$Ni_{11}(HPO_3)_8(OH)_6$	232	42	1.60	NF	*Energy Environ. Sci.* **2018**, *11*, 1287
NiFe MOF	240	87	1.56	NF	*Adv. Energy Mater.* **2018**, DOI: 10.1002/aenm.201801065
N-Ni_3S_2	N.A.	110	1.48	NF	*Adv. Mater.* **2017**, *29*, 1701584
FeCoCH	228	77	1.45	NF	*Adv. Energy Mater.* **2018**, DOI: 10.1002/aenm.201800175
Fe-doped CoP	230	98	1.59	NF	*Small* **2018**, *14*, 1704233
